# *Tokorhabditis* n. gen. (Rhabditida, Rhabditidae), a comparative nematode model for extremophilic living

**DOI:** 10.1038/s41598-021-95863-1

**Published:** 2021-08-13

**Authors:** Natsumi Kanzaki, Tatsuya Yamashita, James Siho Lee, Pei-Yin Shih, Erik J. Ragsdale, Ryoji Shinya

**Affiliations:** 1 Kansai Research Center, Forestry and Forest Products Research Institute (FFPRI), Kyoto, Kyoto 612-0855 Japan; 2 School of Agriculture, Meiji University, Kawasaki, Kanagawa 214-8571 Japan; 3 Lulu and Anthony Wang Laboratory of Neural Circuits and Behavior, The Rockefeller University, 1230 York Avenue, New York, NY 10065 USA; 4 Zuckerman Mind Brain Behavior Institute, Columbia University, New York, NY 10027 USA; 5 Department of Ecology, Evolution and Environmental Biology, Columbia University, New York, NY 10027 USA; 6 Department of Biology, Indiana University, 915 E. 3rd Street, Bloomington, IN 47405 USA

**Keywords:** Evolution, Zoology

## Abstract

Life in extreme environments is typically studied as a physiological problem, although the existence of extremophilic animals suggests that developmental and behavioral traits might also be adaptive in such environments. Here, we describe a new species of nematode, *Tokorhabditis* *tufae*, n. gen., n. sp., which was discovered from the alkaline, hypersaline, and arsenic-rich locale of Mono Lake, California. The new species, which offers a tractable model for studying animal-specific adaptations to extremophilic life, shows a combination of unusual reproductive and developmental traits. Like the recently described sister group *Auanema*, the species has a trioecious mating system comprising males, females, and self-fertilizing hermaphrodites. Our description of the new genus thus reveals that the origin of this uncommon reproductive mode is even more ancient than previously assumed, and it presents a new comparator for the study of mating-system transitions. However, unlike *Auanema* and almost all other known rhabditid nematodes, the new species is obligately live-bearing, with embryos that grow in utero, suggesting maternal provisioning during development. Finally, our isolation of two additional, molecularly distinct strains of the new genus—specifically from non-extreme locales—establishes a comparative system for the study of extremophilic traits in this model.

## Introduction

Extremophilic animals offer a window into how development, sex, and behavior together enable resilience to inhospitable environments. A challenge to studying such animals has been to identify those amenable to laboratory investigation^1,2^. Nematodes have stood out as tractable models for extremophile physiology, given their diversity of environmental responses—including to desiccation, freezing, extreme heat, and extreme pH—as well as, for some clades, their ease of manipulation and their rapid generation times^3–6^. Beyond using individual models to identify genetic correlates and causes of environmental resilience, however, a comparative approach is additionally needed to reveal the order of events giving rise to such resilience during evolution^7^.

A recent survey of an alkaline, hypersaline, and arsenic-rich site (Mono Lake, California, USA) identified an ecological community of nematodes that have independently colonized the lake with various success^8^. Among these nematodes was an undescribed species (represented by strain PS8402) belonging to the paraphyletic group “Rhabditidae,” which includes the celebrated model *Caenorhabditis* *elegans*, in addition to many other species easily cultured in the laboratory^9^. Tentatively assigned to *Auanema*^10^, PS8402 showed some similarities to that genus, most notably trioecy, a reproductive mode consisting of males, females, and self-fertile hermaphrodites. Interestingly, previously described *Auanema* species could, like strain PS8402, withstand high levels of arsenic, indicating this ability to be a preadaptation in PS8402.

However, a unique life-history trait noted for PS8402, with respect to *Auanema*, was that it is viviparous. Vivipary in a broad sense, especially facultative vivipary, is relatively common among rhabditid nematodes, both parasitic and free-living, including *C.* *elegans*. In most cases, such vivipary takes the form of *endotokia* *matricida*, or “bagging,” whereby stressors induce normally oviparous mothers to retain their eggs in the uterus, where the larvae hatch, consume the body contents of the mother, and then emerge through the mother’s body wall^11–14^. Other forms of ovovivipary, which we define here as birth following the intrauterine hatching from an egg from a rigid egg shell (i.e., no embryonic growth), are also present among rhabditid nematodes^15,16^. In contrast, vivipary with embryonic growth is much rarer in nematodes: among “Rhabditidae,” which includes the family Diplogastridae, the latter form of vivipary has only been reported for the diplogastrid genus *Sudhausia*, which comprises associates of dung beetles^17^. Therefore, a description of vivipary in PS8402 is needed to understand the life history of this extremophilic nematode and to determine the functional relationship, if any, between this reproductive strategy and these nematodes’ peculiar lifestyle.

Beyond a full description of PS8402, a phylogenetic infrastructure that includes closely related species is also needed to establish PS8402 as a model for the evolution of extreme lifestyles. To this end, we have identified two other nematode isolates that, together with PS8402, form a clade that is exclusive of *Auanema*. Because these previously unreported nematodes, which are also viviparous, were isolated from sources other than an alkaline, arsenic-rich habitat, they provide the necessary comparative framework for colonization of the latter. Here, we formally describe PS8402 as a new species belonging to a new genus of rhabditid nematodes. Furthermore, we show that PS8402 shows obligate vivipary with embryonic growth in utero and that vivipary evolutionarily predated the colonization of Mono Lake. In our description, we report divergent reproductive (vaginal) morphology associated with vivipary, and we report derived, dispersal stage (dauer) behavior of the new species. In summary, we present morphological, life-history, and phylogenetic data that support the new species as a potential model for extremophilic physiology in nematodes.

## Materials and methods

### Nematode collection and culturing

Nematodes were collected from soil sampled at Mono Lake, CA, USA in August 2016 (37° 56′ 21.90″ N, 119° 1′ 25.93″ W). Nematodes were cultured on nematode growth medium (NGM) seeded with a lawn of *Escherichia* *coli* strain OP50, whereafter it was kept as a laboratory strain with the culture code PS8402. For further molecular analyses, an isogenic line, which was propagated from a single hermaphrodite individual for each of 20 generations, was established for this strain. The original, wild-type culture (i.e., prior to inbreeding) was used for morphological observations and molecular profiles.

### Light microscopic observation and preparation of type specimens

Adult nematodes were collected from a one-week-old culture, whereafter they were heat-killed and fixed in TAF (triethanolamine:formalin:distilled water = 2:7:91) for one week. Fixed material was processed to glycerin using a modified Seinhorst’s method^18^ and mounted in glycerin according to the methods of de Maeseneer and d’Herde^19^. Mounted specimens were used for morphometrics and kept as type material. In addition, live adult nematodes from 1-week-old culture were used for detailed morphological observations following the methods of Kanzaki^20^. All micrographs were obtained using a digital camera system (MC170 HD; Leica, Wetzlar, Germany) and morphological drawings were made using a drawing tube (Eclipse Ni; Nikon, Tokyo, Japan) connected to the microscope.

### Scanning electron microscopy

To obtain adult males for scanning electron microscopic (SEM) observation, two or three dauer larvae were transferred onto each of several NGM plates seeded with *E.* *coli* OP50 and incubated at 20 °C. In the next generation, males were present at relatively high frequency and were collected into M9 buffer. Males were killed by heating on a hot plate (60 °C for 5 min) and were pre-fixed in 2% formaldehyde plus 2.5% glutaraldehyde in M9 buffer for 15 h at 4 °C. Nematodes were then post-fixed with 1% osmium tetroxide for 1.5 h and then dehydrated with an ethanol series (25, 50, 70, 80, 90, and twice in 99.5% ethanol). After dehydration, samples were immersed in 100% tert-butyl alcohol twice (15 min each) and then dried with a freeze dryer (JFD-310; JEOL, Tokyo, Japan). Samples were coated with osmium with an sputter coater (HPC-1SW; Vacuum Device, Ibaraki, Japan), and then observed with a SEM (JSM-6700F; JEOL).

### Molecular profiles and phylogeny

To establish molecular profiles and vouchers for the new species, approximately 4 kb of ribosomal DNA (rDNA), including nearly the full length of small subunit (SSU), intergenic transcribed spacer (ITS), and D1–D4 extension segments of the large subunit (LSU), were sequenced for the new species and another, undescribed species (coded as NKZ329). In addition to these two species, a partial SSU (*ca*. 0.9 kb of 5′ end) was determined for a second undescribed species (EJR13) for inclusion in phylogenetic analysis. All sequences were derived from bulk DNA samples, which were prepared from cultured nematodes (*ca*. 20 individuals) following methods described by Kikuchi et al*.*^21^ and Tanaka et al*.*^22^, and sequences were determined with PCR-direct sequencing according to the method of Ekino et al*.*^23^, i.e., a long (*ca.* 4 kb) fragment was amplified with a primer set SSU988F (5′-CTC AAA GAT TAA GCC ATG C-3′) and D4R (5′-GCG GTA TTT GCT ACT ACC AYY AMG ATC TGC-3′), and the sequences determined using amplified primers and internal primers, SSUF22 (5′-TCC AAG GAA GGC AGC AGG C-3′), SSUF23 (5′-ATT CCG ATA ACG AGC GAG A-3′), SSU1813F (5′-CTG CGT GAG AGG TGA AAT-3′), SSUR13 (5′-GGG CAT CAC AGA CCT GTT A-3′), SSUR22 (5′-GCC TGC TGC CTT CCT TGG A-3′), SSU1912R (5′-TTT ACG GTC AGA ACT AGG G-3′), SSU2646R (5′-GCT ACC TTG TTA CGA CTT TT-3′), 18SF (5′-CGT AAC AAG GTA GCT GTA G-3′), 28SR (5′-TTT CAC TCG CCG TTA CTA AGG-3′), IKF1 (5′-GGG TCG ATG AAG AAC GCA G-3′), IKF2 (5′-CTG CGT TCT TCA TCG ACC-3′), D1F (5′-AAG GAT TCC CTT AGT AAC GGC GAT TG-3′), D2a (5′-ACA AGT ACC GTG AGG GAA AGT TG-3′), D2aR (5′-CAA CTT TCC CTC ACG GTA CTT GT-3′), D3b (5′-TCG GAA GGA ACC AGC TAC TA-3′), D3a (5′-GAC CCG TCT TGA AAC ACG GA-3′), and D3aR (5′-TCC GTG TTT CAA GAC GGG TC-3′) for the former two species, and only SSU988F and SSU1912R were used for EJR13.

Phylogenetic relationships of the new species to other rhabditid nematodes were inferred from partial SSU sequences by Bayesian analysis. First, sequences were aligned using MAFFT^24,25^ under default settings. Base-substitution models for each gene were determined using the Akaike information criterion (AIC) as implemented in MEGA 6^26^. Combined Bayesian analysis was performed using MrBayes 3.2^27,28^; four chains were run for 4 × 10^6^ generations, and Markov chains were sampled at intervals of 100 generations^29^. Two independent runs were performed, and after confirming the convergence of runs and discarding the first 2 × 10^6^ generations as burn-in, the remaining topologies were used to generate a 50% majority-rule consensus tree. Species were included in the analysis as informed by Kanzaki et al*.*^10^. Accession numbers of included sequences are given in Supplementary Table S1.

### Fecundity measurements

L4 larvae of the new species were singled onto NGM plates seeded with *E.* *coli* OP50 and allowed to develop for 24 h into day-1 adults. Adults were then transferred daily onto new seeded plates until they died, and the progeny that they laid were counted every day. Ten replicates were tested. Animals were maintained at 22.5 °C, and adults were transferred with an eyelash to minimize damage from picking.

### Sex ratios of self-progeny

To study reproductive mode in the new species, we quantified sex ratios among offspring produced by a single hermaphrodite. First, we established that all dauers developed into adult hermaphrodites, as in *Auanema* species^30^. Therefore, dauer juveniles were selected to become the mothers from which offspring sexes were quantified. Each individual dauer juvenile was placed on an NGM medium (40 mm diam.) seeded with *E.* *coli* OP50 and kept in a culture room (approximately 23 °C), after which the nematode was examined for developmental stage and reproduction twice a day (approximately every half-day) until it died. As soon as reproduction commenced, each mother was transferred to a new culture plate, also twice a day. The sex was determined as male *vs*. morphological female (i.e., female/hermaphrodite) for each offspring left on each plate from which the mother was removed. Specifically, sex was determined once larvae had reached the L3 stage or L4, according to body shape and shape of genital *anlagen*, as observed under a dissecting microscope. Male juveniles were then removed from the culture medium, while the rest of the juveniles (females/hermaphrodites) were allowed to proceed with development. After at least one day of adulthood, the sex of each individual was distinguished according to the contents of their uteri: individuals carrying only undeveloped oocytes were detetermined to be females, whereas hermaphrodites carried juvenile offspring in their uterus. Once reproduction ceased, the number of male, female, and hermaphrodite offspring were tallied for each half-day time point. Offspring sexing was performed for six hermaphroditic mothers.

## Results

### Taxonomic description of *Tokorhabditis* n. gen.

Etymology: the genus name is derived from the Greek *τόκος* (“birth”) and “*Rhabditis*,” the type genus of Family Rhabditidae.

Rhabditidae. Diagnostic characters of the genus that are in this combination not found in species from other genera: males much smaller than females/hermaphrodites. Stoma (buccal cavity) tube-like, comprising simple short tube-like cheilo-, gymno-, and stegostom, where stegostom occupies about half of stoma (pharyngeal sleeve present), metastegostom possessing two small denticles on each of its three radial sectors; median bulb relatively well developed, round to square. Vagina of young female/hermaphrodite folded, forming “Z’ shape in lateral view, albeit folding unclear in mature individuals after laying juveniles, and surrounded by layers of flattened, elongated cells stretched longitudinally and perpendicularly from the vagina. Female tail conical; bursa on male tail open anteriorly with more or less distinctly bilobed posterior margin; eight genital papillae (GP) and pore-like phasmids posterior to genital papillae. One to two GP precloacal, 5th and 7th attached to the dorsal surface of the fan. Anterior pairs forming papilliform GP or short bursal rays. 6th GP separate, 7th and 8th close together. Spicules separate and dagger-shaped; gubernaculum half as long as spicules. Dauer juveniles ensheathed. Three sexes (male, female, self-fertilizing hermaphrodite) present. Males rare, but still found in populations with very low density. Female and hermaphrodite both obligately viviparous (Supplementary Data 1), with embryos growing in size before reaching the L1 stage^8^.

Type species:

*Tokorhabditistufae* n. gen., n. sp.

### Relationships

The adult morphology of *Tokorhabditis* n. gen., including the stoma, male tail characters (papilla configuration and variation therein), and presence of three sexes, overlaps with that of *Auanema*^10^, the closest genus based on biological and morphological characters. However, the new genus is distinguished from *Auanema* most prominently by being obligately viviparous. Additionally, *Tokorhabditis* n. gen. is distinguished by its male phasmid structure, being pore-like vs. papilliform, and by the genitalia of females/hermaphrodites, specifically by the presence of a vaginal folding and by a tissue composed of layered, flattened, elongated cells stretched perpendicularly from the vagina. Diagnostic characters of *Tokorhabditis* n. gen. with respect to phylogenetically close genera *Auanema*, *Rhabditella*, and *Cephaloboides*, as informed by Kiontke and Sudhaus^31^, Scholze and Sudhaus^32^, Sudhaus^33^ and Kanzaki et al*.*^10^, are summarized in Table 1.

**Table 1:** Comparison of key typological characters among four closely related genera.

	*Tokorhabditis*	*Auanema*	*Rhabditella*	*Cephaloboides*	*Haematozoon*	*Rhabditoides*
Mating system	M/F/H	M/F/H	M/F	M/F	M/F	M/F
Reproduction	Viviparous	Oviparous	Oviparous	Oviparous	Oviparous	Oviparous
Bursa	Leptoderan	Leptoderan or peloderan	Leptoderan or absent	Leptoderan	Leptoderan	Leptoderan or vestigial
Genital papillae	8 pairs (GP5, 7 latero-dorsal)	8 pairs (GP5, 7 latero-dorsal)	9 pairs (GP4, 8 latero-dorsal)	9 pairs (GP1, 4, 8 latero-dorsal)	9 pairs (direction not consistent)	9 pairs (direction not consistent)
Male phasmid	Pore-like at the level of GP7-8	Papilliform at root of spike or posterior to all papillae	Papilliform at root of spike	Papilliform at root of spike	Pore-like anterior to GP6	Pore-like posterior to GP8 or 9
Female/hermaphrodite tail	Elongate conoid	Elongate conoid	Very long and elongate conoid	Cupola-shaped	Elongate conoid	Elongate conoid

*Tokorhabditis* is also similar to *Haematozoon* and *Rhabditoides* in general morphology, e.g., with a more or less long and narrow stoma and conspicuous pharyngeal sleeve, conical female/hermaphrodite tail, dagger-shaped male spicule, and a gubernaculum that is wealky arcuate and approximately half of spicule in length^33^. However, *Tokorhabditis* is readily distinguished from both genera with its biological characters, three vs. two sexes, vivipary vs. ovipary, and the number and arrangement of male genital papillae, namely by having eight pairs with one to two that are precloacal vs. nine pairs with three to four precloacal pairs and by the 5th and 7th pairs being dorsally oriented *vs.* laterally oriented^33^.

### *Tokorhabditis tufae* n. gen., n. sp.

**Figure 1:**
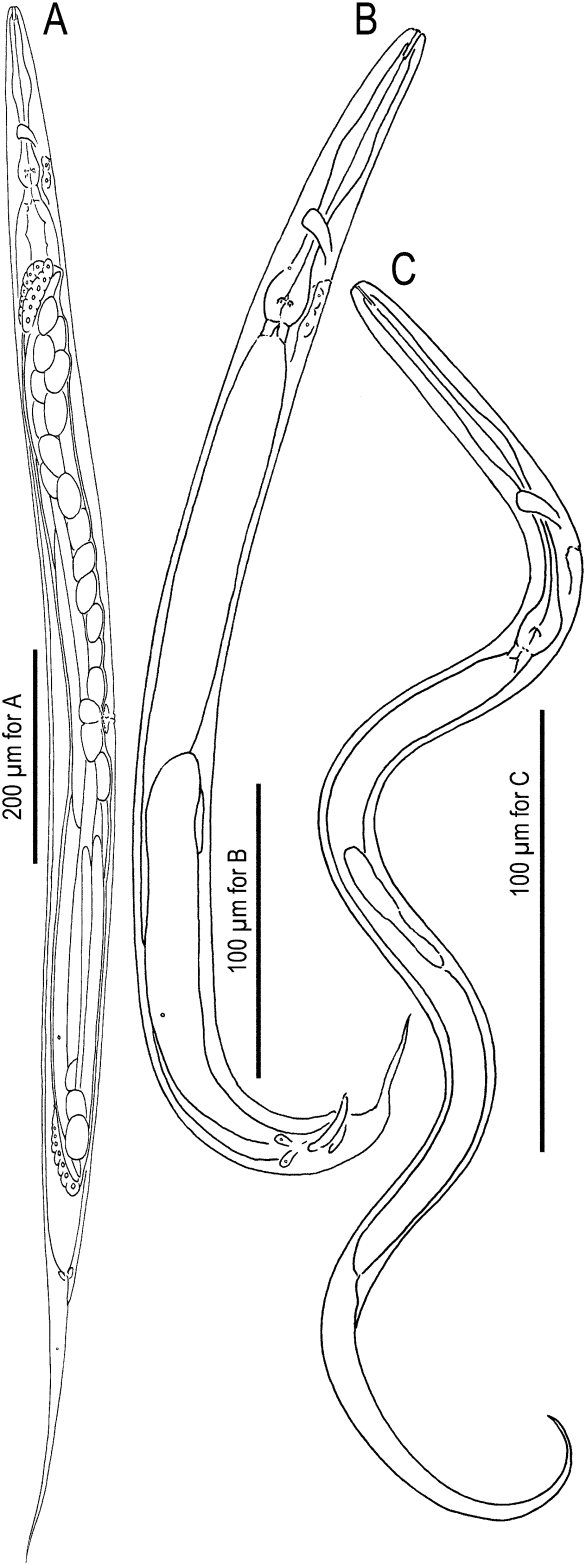
Mature hermaphrodite, male and dauer juvenile of *Tokorhabditis* *tufae* n. gen., n. sp. (**A**) Mature hermaphrodite; (**B**) Male; (**C**) Dauer juvenile.

**Figure 2:**
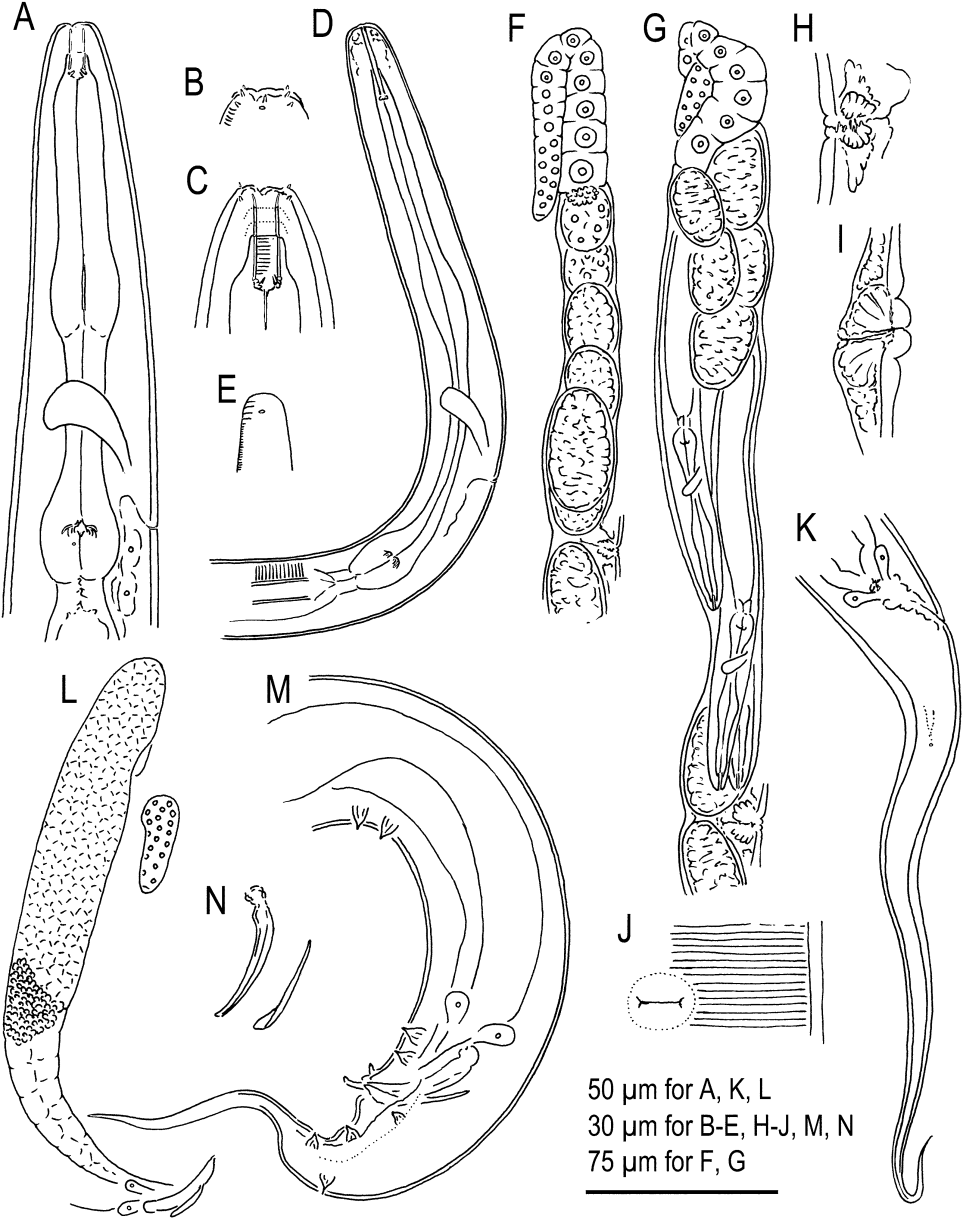
Hermaphrodite, male and dauer juvenile of *Tokorhabditis* *tufae* n. gen., n. sp. (**A**) Anterior part of hermaphrodite in right lateral view; (**B**) Surface of lip region of hermaphrodite; (**C**) Stomatal region of hermaphrodite in right lateral view; (**D**) Anterior part of dauer juvenile in right lateral view; (**E**) Surface of lip region of dauer juvenile; (**F**) Anterior gonad of young hermaphrodite in right lateral view; (**G**) Anterior gonad of mature hermaphrodite in right lateral view; (**H**) Vulval region of young hermaphrodite in left lateral view; (**I**) Vulval region of mature hermaphrodite in right lateral view; (**J**) Ventral view of young hermaphrodite in ventral view; (**K**) Tail region of hermaphrodite in right lateral view; (**L**) Male gonad in right lateral view where reflex part is drawn separately; (**M**) Male tail region in left lateral view; (**N**) Spicule and gubernaculum in left lateral view.

**Figure 3:**
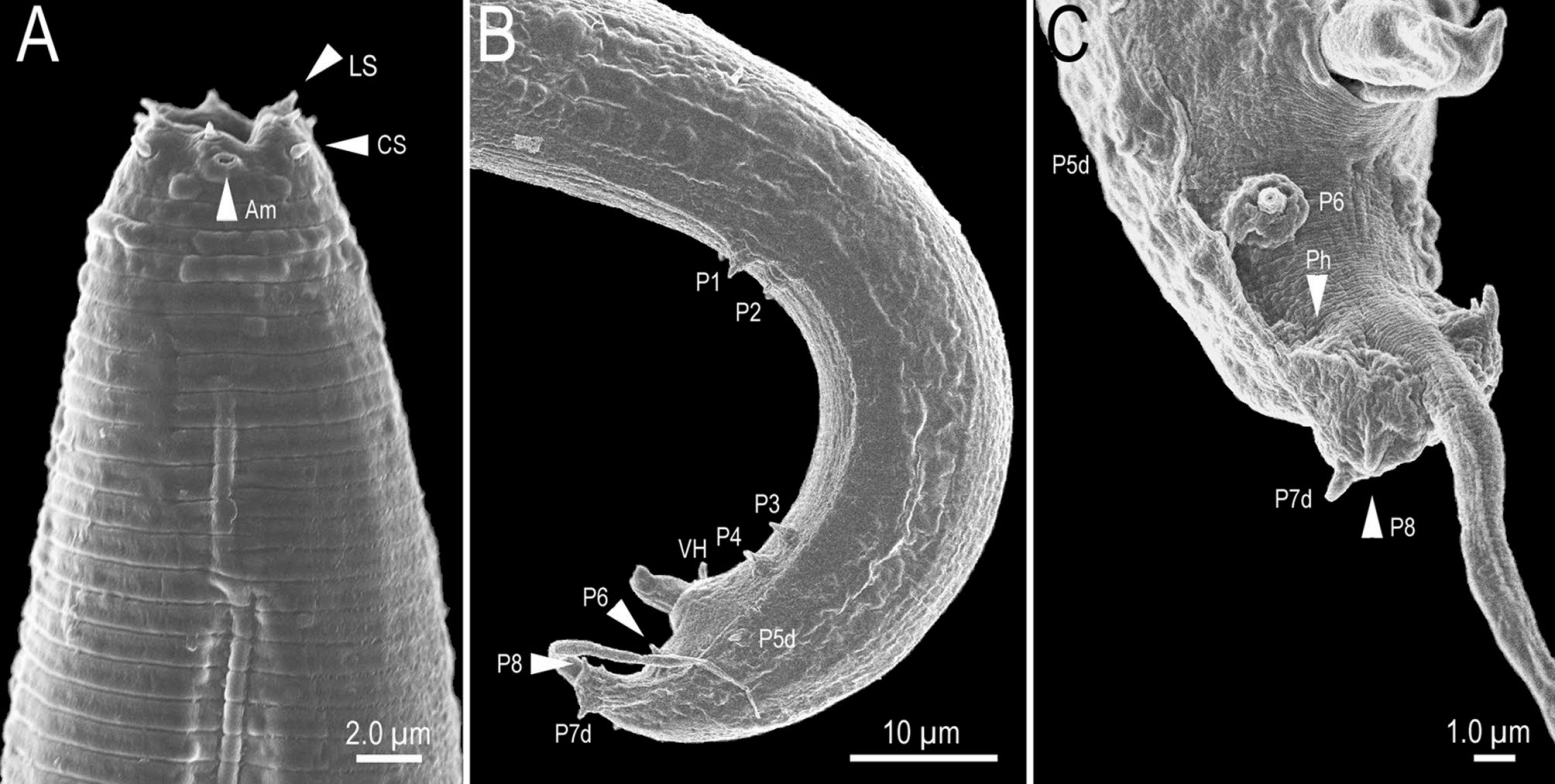
*Tokorhabditistufae* n. gen., n. sp. (**A**) Left lateral view of head region of adult hermaphrodite; (**B**) Left lateral view of whole male tail; (**C**) Male tail tip region in right subventral view. Amphid (Am), labial sensilla (LS), cephalic sensilla (CS), papillae (P + number, and “d” indicates dorsally or laterally opened papillae), ventral precloacal hook (VH) and phasmid (Ph) are labelled.

**Figure 4:**
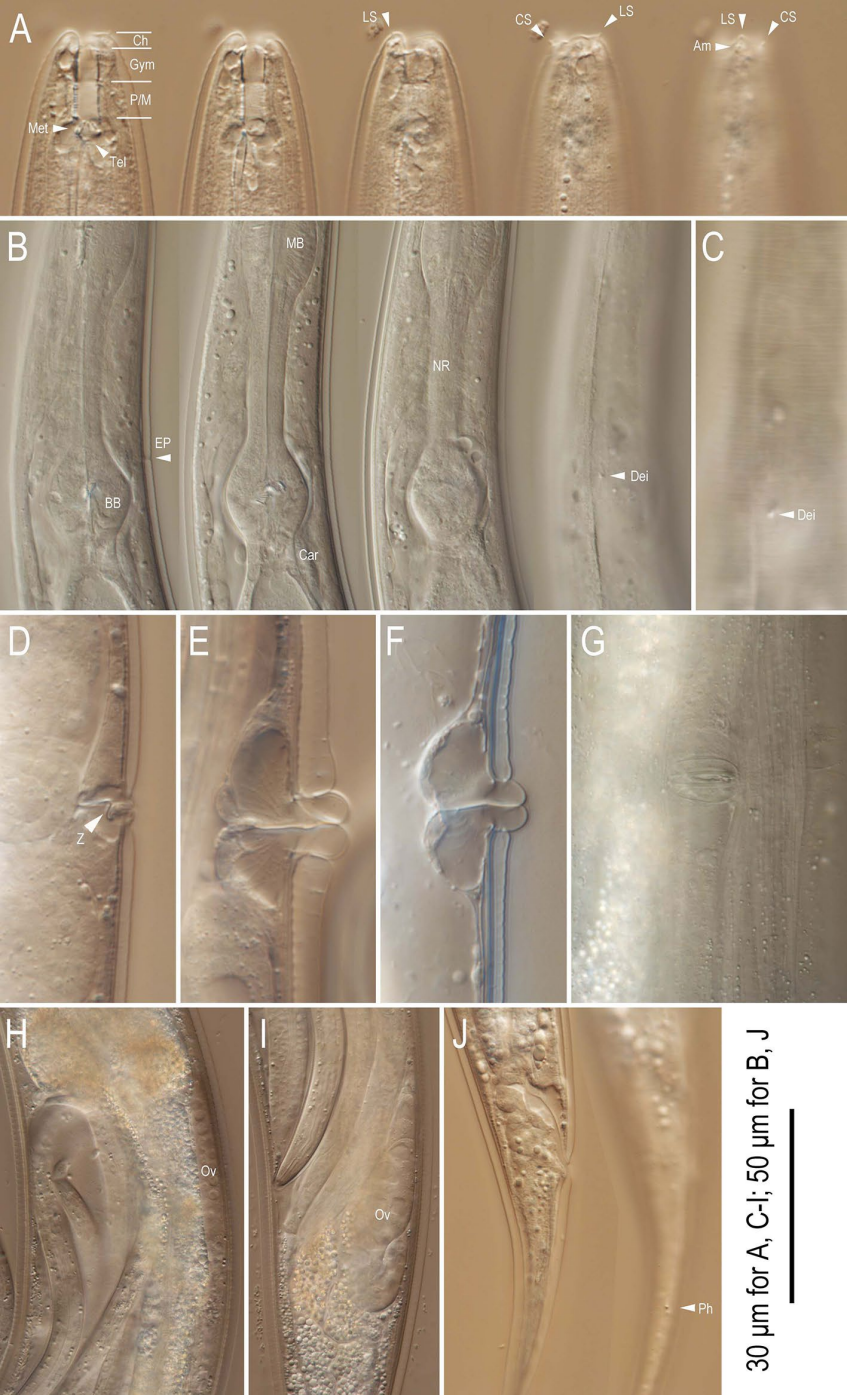
Adult hermaphrodite of *Tokorhabditis* *tufae* n. gen., n. sp. (**A**) Right lateral view of stoma in five different focal planes; (**B**) Right lateral view of pharyngeal region in four different focal planes; (**C**) Body surface of pharyngeal region; (**D**–**F**) Right lateral view of vulval region of very young hermaphrodite (**D**), young hermaphrodite (**E**) and mature hermaphrodite (**F**); (**G**) Ventral view of vulval region; (**H**,**I**) Left lateral view of anterior (**H**) and posterior (**I**) ovaries of mature hermaphrodite; (**J**) Right lateral view of tail in two different focal planes. Abbreviations are as follows: (**A**) *Ch* cheilostom, *Gym* gymnostom, *P/M* pro and mesostegostom, *Met* metastegostom, *Tel* telostegostom, *LS* labial sensilla, *CS* cephalic sensilla, *Am* amphid; (**B**,**C**); *Z* Z-shaped vaginal folding (**D**); *MB* median bulb, *BB* basal bulb, *EP* secretory-excretory pore, *NR* nerve ring, *Dei* deirid; (**H**,**I**) *Ov* ovary; (**J**) *Ph* phasmid.

**Figure 5:**
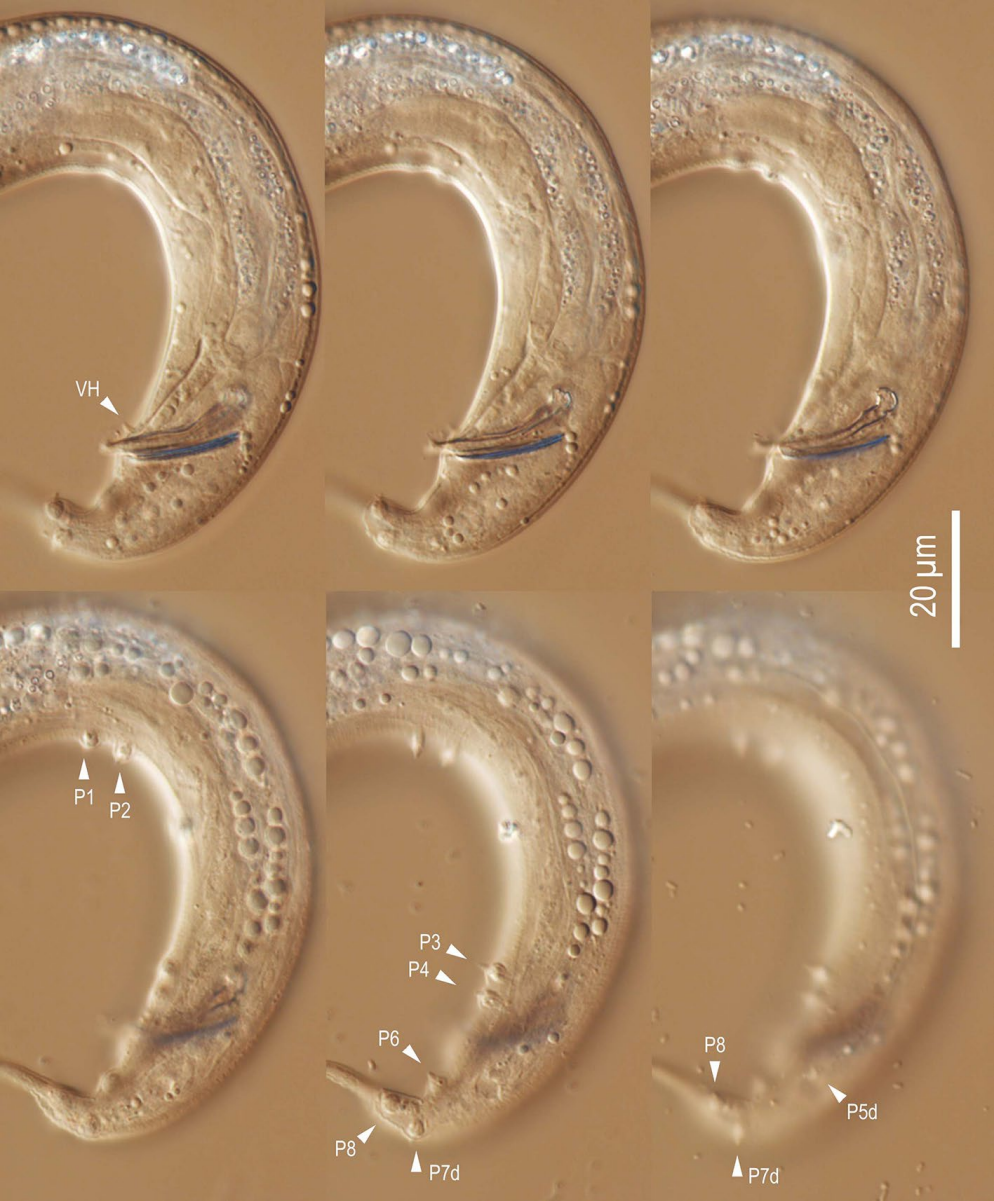
Left lateral view of male tail of *Tokorhabditis* *tufae* n. gen., n. sp. in six different focal planes. Abbreviations are as follows: *P + number* genital papillae where “d” indicates dorsally opened papillae, *VH* ventral precloacal hook.

**Figure 6:**
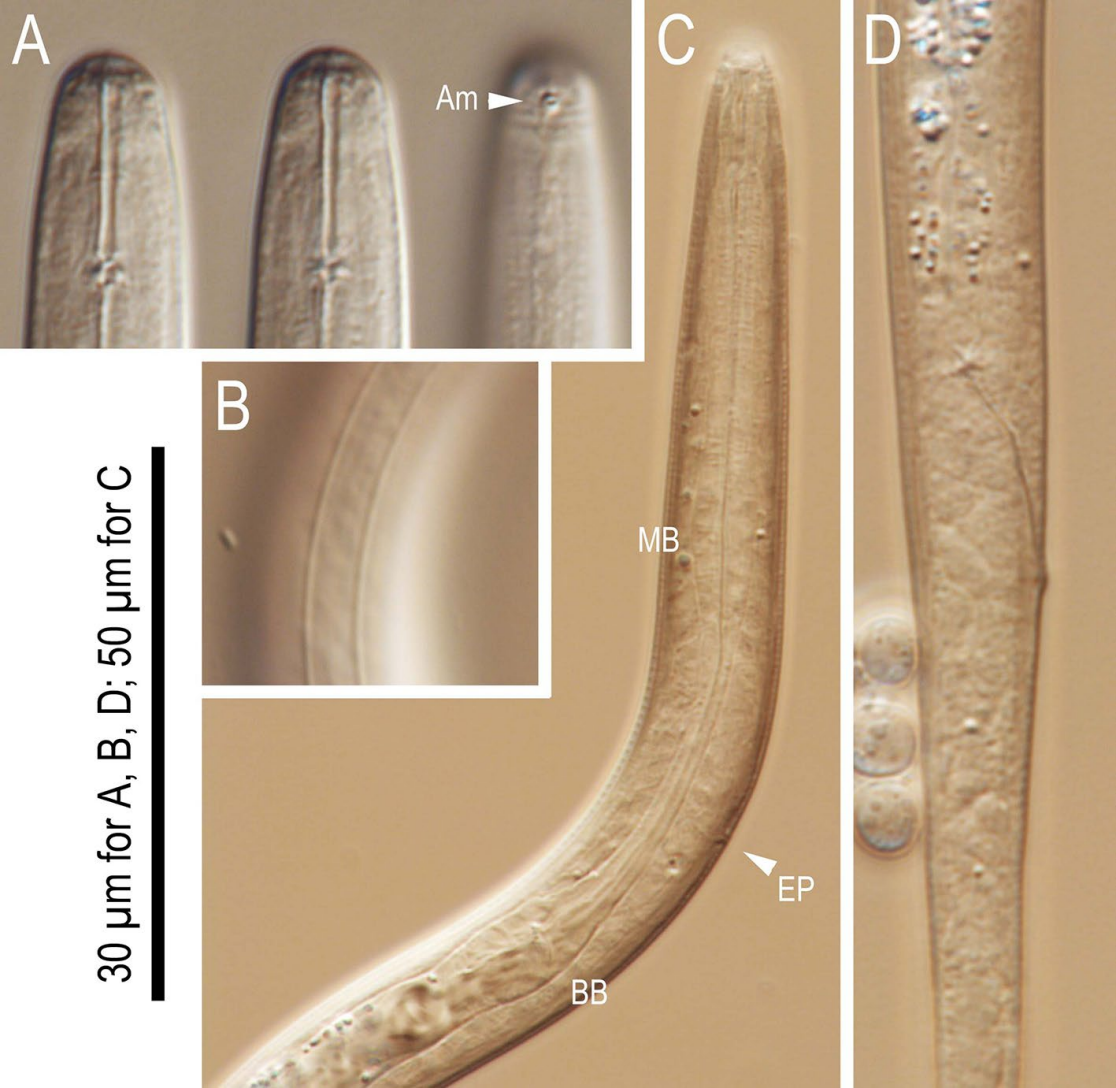
Dauer juvenile of *Tokorhabditis* *tufae* n. gen., n. sp. (**A**) Lip region in three different focal planes; (**B**) Body surface showing lateral field; (**C**) Right lateral view of anterior region; (**D**) Right lateral view of tail region. Sheath of the dauer was removed manually for observation.

Figures 1, 2, 3, 4, 5, 6; Supplementary Data 2.

The species epithet is the Latin genitive of “tufa,” which describes the carbonate mineral precipitates characteristic of the majestic rock towers of Mono Lake.

### Measurements

See Table 2.

**Table 2:** Morphometric values for *Tokorhabditis* *tufae* n. gen., n. sp.

	Holotype male (#6)	Paratype males	Paratype hermaphrodites	Paratype females	Paratype dauers
n	–	9	10	10	10
L	448	449 ± 25 (406–489)	954 ± 123 (798–1135)	770 ± 39 (716–825)	352 ± 6.9 (336–361)
a	17.6	18.5 ± 1.6 (16.4–21.3)	15.1 ± 1.2 (13.7–17.0)	17.1 ± 1.3 (14.8–19.4)	28.5 ± 2.8 (23.3–33.2)
b	3.9	4.0 ± 0.2 (3.9–4.4)	6.9 ± 0.6 (6.1–7.8)	6.0 ± 0.3 (5.6–6.4)	3.9 ± 0.1 (3.8–4.0)
C^a^	8.4	9.1 ± 0.6 (8.2–10.2)	5.9 ± 0.5 (5.1–6.6)	4.3 ± 0.1 (4.1–4.5)	4.3 ± 0.3 (4.1–4.9)
c′^a^	3.3	3.1 ± 0.2 (2.7–3.4)	8.7 ± 0.5 (7.5–9.2)	10.4 ± 0.7 (9.1–11.3)	11.2 ± 0.9 (9.7–12.7)
T^b^ or V	40.7	40.9 ± 5.1 (29.4–48.2)	47.2 ± 0.8 (45.9–48.2)	44.2 ± 0.6 (42.8–45.0)	–
Maximum body diam^c^	25.5	24.4 ± 2.6 (20.9–28.6)	63 ± 7.8 (50–74)	45 ± 4.3 (40–54)	12.5 ± 1.3 (10.7–15.3)
Stoma diam	2.6	2.3 ± 0.4 (1.5–2.8)	3.9 ± 0.3 (3.6–4.1)	4.2 ± 0.2 (4.1–4.6)	–
Stoma depth	12.8	12.3 ± 0.7 (11.2–13.3)	15.4 ± 1.1 (13.8–16.8)	13.9 ± 0.4 (13.3–14.3)	–
Stoma depth/diam. ratio	5.0	5.6 ± 1.0 (4.4–8.0)	4.0 ± 0.4 (3.5–4.7)	3.3 ± 0.2 (3.0–3.5)	–
Anterior pharynx length	45	45 ± 1.6 (42–46)	60 ± 3.2 (57–67)	56 ± 0.9 (54–57)	40 ± 1.9 (37–43)
Posterior pharynx length	53	52 ± 2.1 (49–56)	59 ± 4.6 (50–64)	56 ± 0.8 (54–57)	37 ± 1.3 (35–39)
Anterior/posterior pharynx length ratio	0.85	0.87 ± 0.04 (0.77–0.94)	1.04 ± 0.11 (0.92–1.33)	1.00 ± 0.02 (0.97–1.03)	1.08 ± 0.08 (0.97–1.25)
Median bulb diam	10.7	10.3 ± 0.7 (9.2–11.2)	18.2 ± 1.4 (16.3–20.4)	15.7 ± 0.7 (14.7–16.8)	5.6 ± 0.4 (5.1–6.6)
Basal bulb diam	16.8	15.2 ± 1.4 (13.3–17.3)	21.5 ± 1.3 (19.4–23.7)	18.6 ± 1.2 (17.8–21.4)	6.9 ± 0.5 (6.6–8.2)
Nerve ring from anterior end	72	71 ± 3.0 (64–75)	94 ± 4.1 (87–98)	84 ± 1.3 (81–86)	55 ± 2.2 (51–58)
Secretory-excretory pore from anterior end	90	91 ± 5.6 (79–102)	119 ± 12.6 (102–139)	105 ± 4.1 (100–116)	66 ± 1.5 (65–69)
Cloacal or anal body diam	16.3	15.7 ± 1.0 (14.3–17.9)	18.6 ± 1.4 (16.8–20.4)	17.1 ± 1.4 (14.8–18.9)	7.3 ± 0.7 (6.1–8.7)
Tail length^a^	54	49 ± 3.3 (44–54)	162 ± 8.4 (150–173)	178 ± 10.7 (167–200)	82 ± 4.9 (71–86)
Tail spike length	22.4	19.5 ± 2.0 (15.8–22.4)	–	–	–
Whole gonad length	182	183 ± 18 (144–210)	–	–	–
Reflex part of testis	27	28 ± 6.7 (18–39)	–	–	–
*Vasdeferens* length^b^	63	64 ± 7.7 (50–74)	–	–	–
% of *vas* *deferens* to whole gonad	34.6	35.2 ± 6.3 (28.1–51.5)	–	–	–
Spicule length (curve)	23.0	23.1 ± 1.8 (20.4–26.5)	–	–	–
Spicule length (chord)	20.4	21.0 ± 1.7 (18.9–24.0)	–	–	–
Gubernaculum length (chord)	14.3	14.6 ± 0.9 (13.8–16.8)	–	–	–
Anterior ovary length	–	–	122 ± 17 (97–150)	88 ± 13 (65–103)	–
Posterior ovary length	–	–	120 ± 12 (102–138)	88 ± 14 (65–107)	–
Anterior/posterior ovary length ratio	–	–	1.02 ± 0.12 (0.74–1.22)	1.00 ± 0.07 (0.92–1.14)	–
Phasmid from anus	–	–	30.4 ± 1.9 (26.5–33.2)	25.6 ± 2.3 (23.0–30.1)	–
Relative position of phasmid to anal body diam^d^	–	–	1.63 ± 0.12 (1.49–1.91)	1.49 ± 0.12 (1.31–1.69)	–
Relative position of phasmid to tail length^e^	–	–	18.8 ± 1.1 (17.2–21.2)	14.3 ± 1.2 (12.8–16.5)	–

^a^Tail length including tail spike.

^b^Gonad length including reflex part and *vasdeferens.*

^c^Body diam. is maximum at vulval part in hermaphrodite and female (vulval body diam. = maximum body diam.).

^d^Calculated as anus-phasmid distance/anal body diam.

^e^Calculated as 100 × anus-phasmid distance/whole tail length.

### Description

#### Adults

Body cylindrical. Cuticle thick, annulation fine, greatest annule width being 1.5–2.0 μm. Lateral field present, without ridges (alae). Lip region continuous with body contour, separated into six sectors of equal size. Stomatal opening beset by three pairs of lip sectors (two subdorsal sectors; right lateral plus subventral sectors; left lateral plus subventral sectors), forming triangular stomatal (buccal cavity) opening *en* *face*. Labial sensilla setiform, one per lip sector. Cephalic sensilla setiform, one per dorsal sector and subventral sector (four total). Amphid openings at the level of margin between cheilo- and gymnostom, forming oval-shaped pores. Stoma cylindrical; cheilostom, gymnostom, and stegostom with approximately 1:2:3 of relative length, respectively such that pharyngeal sleeve (= stegostom) surrounds posterior half of stoma. Cheilostom and gymnostom forming short tube, with arcade syncytia slightly visible where they meet gymnostom. Prostegostom and mesostegostom forming a simple short cylinder surrounded by pharyngeal sleeve, and comprising about 55% of stomatal cylinder. Metastegostom slightly anisotopic and isomorphic with two small denticles on each sector, slightly more posterior on the dorsal side. Procorpus muscular, cylindrical. Metacorpus muscular, forming a well-developed median bulb. Isthmus slender, not muscular. Basal bulb rounded (not polygonal) and possessing weakly-developed duplex haustrulum posterior to valves. Anterior pharynx (Procorpus plus metacorpus) slightly longer than posterior pharynx (isthmus plus basal bulb). Cardia (pharyngo-intestinal junction) well-developed, funnel-shaped. Nerve ring circling middle of isthmus. Secretory-excretory pore conspicuous, slightly variable in position among individuals, mostly overlapping with level of basal bulb. Excretory duct extending anteriad from pore, then reflexing posteriad distally. Excretory cell adjacent to excretory duct, slightly posterior to pore. Deirid on lateral side of body, around level of excretory pore.

#### Males

Two separate lines of lateral field sometimes observed, i.e., not conspicuous, with visibility depending on preparation of specimens. Tail region ventrally curved when killed by heat. Stoma on average six times as long as wide. Testis single, on the right of intestine; anterior part ventrally reflexed. Distal third of gonad forming *vas* *deferens*, either empty or containing small sperm cells. Two subventral glands and one dorsal cloacal (anal) gland visible at level of anterior end of retracted spicules. Spicules paired, arranged as a “V” shape in ventral view; often protracted in heat-killed specimens. In lateral view, spicule with roundish-square manubrium separated from blade with constriction; blade widest at just posterior to constriction, then smoothly tapered to bluntly pointed tip; spicule with small but pronounced dorsal, spike-like projection present at slightly anterior to proximal of spicule tip. Gubernaculum short, narrow, slightly ventrally arcuate in lateral view, approximately 2/3 of spicule in length; thin, flat extensions of both sides cover the dorsal side of spicule blade; elongate oval in ventral view. Bursa present, anteriorly open; leptoderan, somewhat polygonal, with smooth edges; distal end of bursa deeply notched, forming a rounded flap on each sides of a slender tail spike with approximately 1.5 times of cloacal body diam. In length. Eight pairs of genital papillae (GP) arranged as < (GP1, GP2), (GP3, GP4), (GP5d, CO), GP6, (phasmid, GP7d, GP8) > , where CO is the cloacal opening, GP1-GP4 form papilliform genital papillae, and other papillae form bursal rays. First four subventral papillae form two doublets (GP1 + GP2 and GP3 + GP4), where first and second doublets located *ca*. 2.5 and 0.5 cloacal body diameters (CBD) anterior to CO; GP5d adcloacal, GP6 between CO and root of tail spike; phasmid, GP7d and GP8 close to each other around the slightly anterior to the root of tail spike. Tips of GP5d and GP7d attached to the dorsal side of bursa, all other GP attached ventrally. Pore-like phasmids with ventral openings in a terminal position near tail tip.

#### Females and hermaphrodites

Females and hermaphrodites are morphologically indistinguishable. Body weakly, smoothly, and ventrally arcuate when heat-relaxed. Vulva located at mid body, forming horizontal slit 1/3 of vulval body diam. In length; cuticle around the vulva lacks annulations. Stoma 3.5–4.7 times as long as wide. Two gonads, one extending anteriad from vulva along right side of body, the other extending posteriad from vulva and along left side, are both dorsally reflexed. Germ cells arranged in multiple (two to three) rows in distal half of ovary, with a transition to single row of well-developed oocytes arranged in proximal half; oocytes nearest oviduct/uterus are most developed in size and most opaque in cell contents. Oviduct not distinct from uterus; spermatheca at boundary between ovary (or, in hermaphrodites, ovotestis) and oviduct/uterus. Spermatheca not clearly separable from rest of reproductive tract, and is distinguished only by presence of sperm (small, rounded cells) rather than structure of reproductive tract. Uterus a long, thick-walled tube between spermatheca and vulva/vagina and clearly expands when carrying well-developed embryos and juveniles. Dorsal wall of the junction of anterior/posterior uterus thickened. Vagina approximately perpendicular to body surface, folded forming ‘Z’ shape in young adults, although folding unclear in mature individuals after laying juveniles, and possessing thick wall, surrounded by a tissue of flattened, elongated cells layered longitudinally, constricted by sphincter muscle at vaginal-uterine junction. Young females/hermaphrodites carrying usually none or only one embryo in each uterus, in old individuals, many (more than 10) well-developed embryos and juveniles are present in an expanded uterus, rendering other gonadal structures vague. Two subventral glands and one dorsal rectal gland observed surrounding intestine-rectum junction and anterior part of rectum. Rectum approximately same as anal body diam. Anus a short horizontal slit at surface; posterior anal lip expands slightly in lateral view. A pair of phasmids located laterally at 1.3–1.9 anal body diam. Or 13–21% of tail length posterior to anus. Tail forming elongate conoid, smoothly tapered to finely elongated conical tip but not filiform.

#### Dauer juveniles

Actively move around on substrate (Supplementary Data 2). Body cylindrical, straight or weakly ventrally arcuate when heat-relaxed. Cuticle thin, smooth, coarsely and shallowly annulated, with two lines of conspicuous lateral field. Anterior end dome-shaped, continuous with body. Amphids with oval-shaped openings, conspicuous, at the level of the posterior end of cheilostom. Labial sensilla observed as refractive dots, inconspicuous. Initially ensheathed in coarsely annulated J2 cuticle; border between J2 cuticle and dauer body transparent. Stoma narrow, approximately 10 times deeper than diam., cylindrical, weakly sclerotized, anterior end closed; separations among cheilostom, gymnostom, and stegostom not clear, but stegostom distinguished by the presence of pharyngeal sleeve. eta- and telostegostom, where stoma meets pharyngeal lumen, more sclerotized than anterior regions of stoma. Procorpus cylindrical, not well-developed, occupying less than 1/3 of corresponding body diam. Metacorpus slightly expanded to form median bulb. Isthmus slightly slenderer than procorpus. Posterior end of corpus (pharynx) forms weakly developed basal bulb with weakly developed duplex haustrulum, smoothly connected to cardia. Procorpus plus metacorpus (anterior pharynx) slightly longer than isthmus plus basal bulb (posterior pharynx). Cardia funnel-shaped, seemingly closed. Nerve ring not well-developed, surrounding the middle part of isthmus. Excretory pore conspicuous, on ventral side of body, at level of middle to posterior parts of isthmus. Excretory tube extending anteriorly, and then reflexing posteriorly. Excretory cells sometimes observed, but not always clear. Genital *anlagen* visible ventrally at mid-body; cells linearly arranged, but number of cells not clearly observed. Two subventral glands and one dorsal gland observed at intestine-rectum junction and anterior part of rectum. Rectum approximately same as anal body diam. In length. Tail more or less elongate conoid with pointed tip.

#### Type habitat and locality

The type strain, PS8402, was originally collected from soil sampled at the bank of Mono Lake, CA, USA (37° 56′ 21.90″ N, 119° 1′ 25.93″ W) in August 2016.

#### Type material

Type specimens include a holotype male, nine paratype males, 10 hermaphrodites, 10 paratype females, and 10 dauer juveniles and deposited in the USDA Nematode Collection (USDANC), Beltsville, MD, USA and in the Swedish Natural History Museum, Stockholm, Sweden. The collection IDs are as follows: the holotype male (T-762t), four paratype males (T-5168p to T-5171p), five paratype hermaphrodites (T-5182 to T-8186), five paratype females (T-5172p to T-5176p), and five dauer juveniles (T-5177p to T-5181p) deposited in USDANC, and five paratype males (SMNH Type-9314–9318), five paratype hermaphrodites (9319–9323), five paratype females (9324–9328), and five dauer juveniles (9329–9333) deposited in the Swedish Museum of Natural History. In addition, several mounted and unmounted specimens of males, females and hermaphrodite specimens are deposited in Kansai Research Center, FFPRI (NK).

### Fecundity of live-bearing hermaphrodites

**Figure 7:**
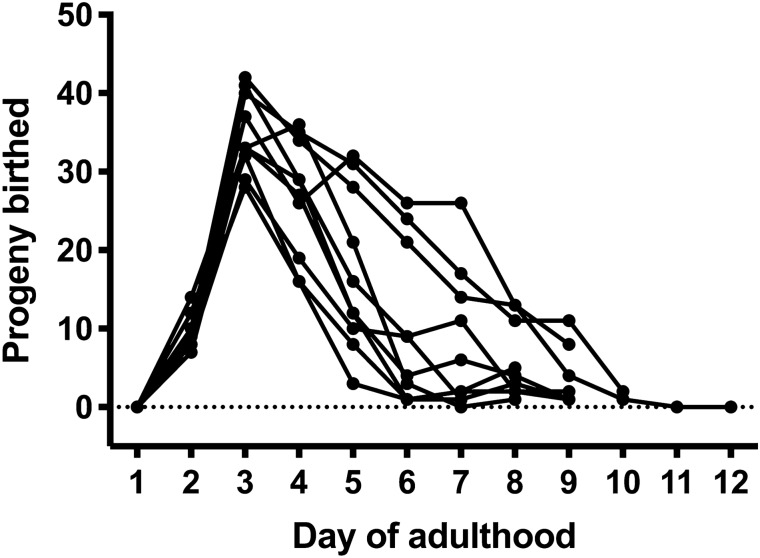
Fecundity of live-bearing hermaphrodites. Lines indicate progeny produced by individual adults, with points representing progeny counts. Line ends indicate the death of the adult.

Selfing hermaphrodites of *T.* *tufae* n. gen., n. sp. produce an average of 115 progeny ± 14 (std. err.) over their reproductive lifespan (Fig. 7). Hermaphrodites produced offspring over an average of 9 days of adulthood, with reproduction fully ceasing only at death. Peak birth rates occur on day 3 of adulthood, with an average of 1.5 births per hour. Overall birth rate over the reproductive lifespan of PS8402 is 0.5 births per hour. We then compared these valus to the mean brood size of the laboratory nematode *C.* *elegans* (327 offspring)^34^ and that of the more closely related nematode *Auanema* *rhodensis* (377 offspring)^35^ (Kruskal–Wallis ANOVA, χ^2^ = 24.7, *p* = 4.42 × 10^–6^, df = 2; Kolmogorov–Smirnov, *p* = 1.71 × 10^–5^ for *T.* *tufae* n. gen., n. sp. and *C.* *elegans* and *p* = 7.32 × 10^–7^ for *T.* *tufae* n. gen., n. sp. and *A.* *rhodensis*).

### Sex ratios of self-progeny of *T. tufae* n. gen., n. sp.

**Figure 8:**
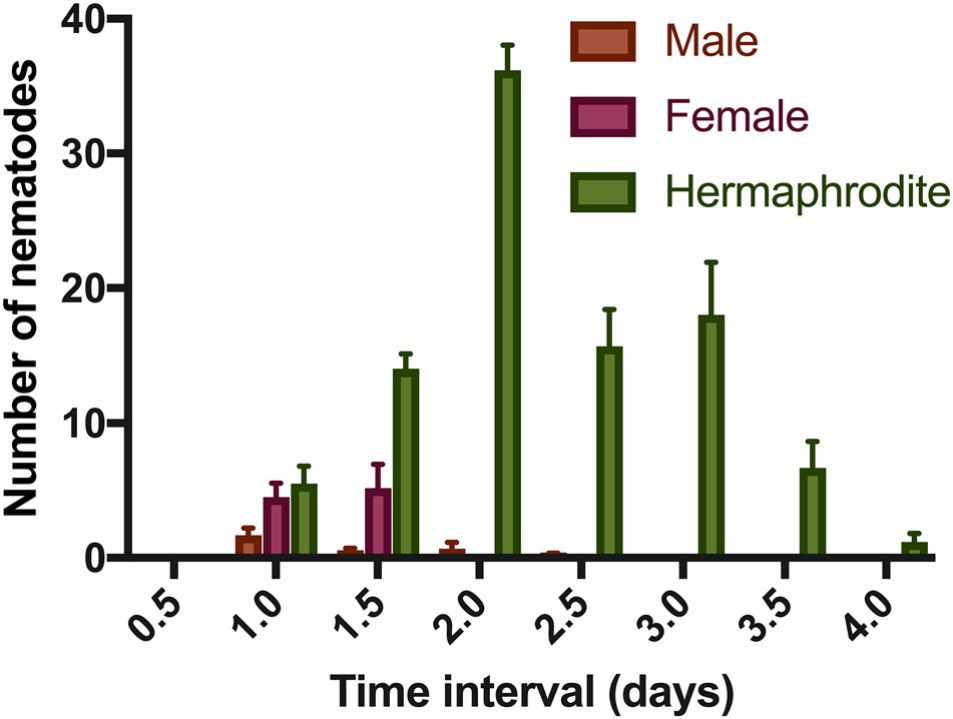
Sex ratios of offspring of a selfing hermaphrodite. Error bars indicate the standard error.

Sexes of offspring from selfing hermaphrodites were determined throughout the mother’s lifespan (Fig. 8). We found the first offspring 1 day after an individual dauer juvenile was placed on a *E.* *coli* OP50 plate. The sex ratio of an entire brood born from a self-cross was 2.7% male, 8.8% female, and 88.5% hermaphrodite. Further, mothers produced female and male offspring only at the beginning of their reproductive lifespan, such that almost all females and males were produced within 2 days of their mothers becoming adults. Hermaphrodites were born throughout the reproductive lifespans of their mothers.

### Phylogenetic relationships of *T. tufae* n. gen., n. sp.

**Figure 9:**
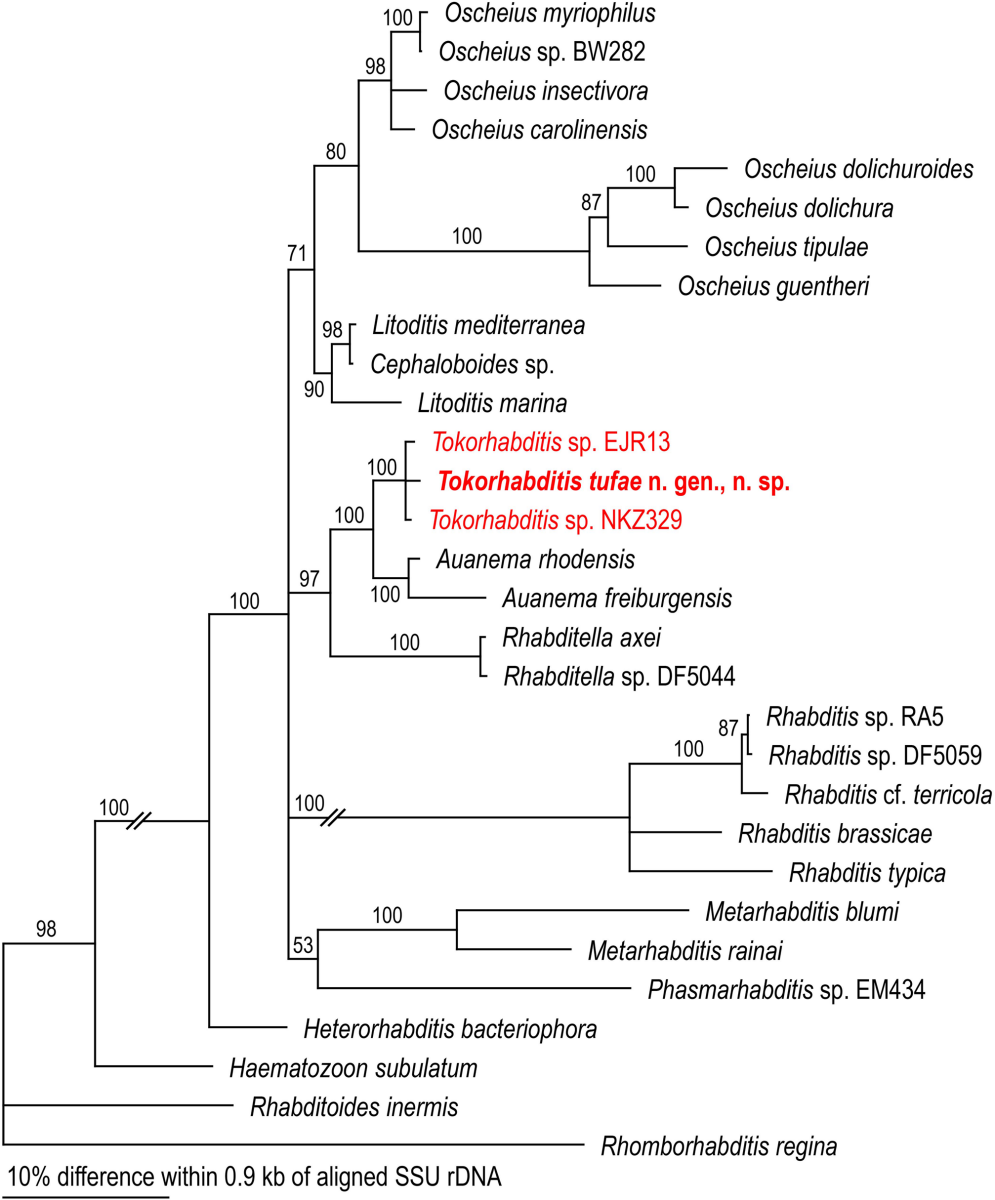
Phylogenetic status of *Tokorhabditis* n. gen. as inferred from SSU under GTR + G + I model (AIC = 13,794.546). Analytical parameters are: lnL = -8592.382; freqA = 0.27, freqC = 0.20. freqG = 0.25, freqT = 0.28; R(a) = 0.87, R(b) = 2.91, R(c) = 2.48, R(d) = 1.00, R(e) = 3.95, R(f) = 1.00; Pinva = 0.25; Shape = 1.16). Posterior probability values exceeding 50% are given on appropriate clades.

Molecular phylogenetic analysis of the partial (*ca*. 0.9-kb) SSU rDNA gene inferred *Tokorhabditis* n. gen. to be the sister group of *Auanema* and that these two genera formed a sister group to *Rhabditella* (Fig. 9). Furthermore, each of these three genera was fully supported (with 100% posterior probability) as monophyletic. Further, the new genus was exclusive of the typologically similar genera *Haematozoon* and *Rhabditoides* (Fig. 9).

## Discussion

Our description of *Tokorhabditis* n. gen. presents a model for extreme physiological resilience, specifically one in a group of nematodes (“Rhabditidae”) that are easily manipulated and well used as laboratory organisms. Moreover, the new species provides a new point of comparison for both the evolution of trioecious reproduction and obligate vivipary, life-history characters that may have—as it is easy to speculate, and now feasible to test—fostered the colonization of such extreme niches as those in Mono Lake.

### An unusual form of vivipary in nematodes

The majority of nematodes are oviparous, although some viviparous species have been reported, both in “Rhabditidae” and elsewhere. However, in most cases where this feature has been adequately described, it is apparent that this vivipary, which would be described as ovovivipary as defined above, is simply the retention of eggs wherein embryonic development proceeds as in oviparous species. For example, in parasitic females of the entomoparasitic species *Deladenus* *nitobei*, a non-rhabditid nematode in which vivipary has been characterized, the physical sizes (volumes) of single-celled embryos are equal to those of juveniles that hatch in utero^36^. In contrast, nematodes of *Tokorhabditis* n. gen. grow in size during their embryonic development. This key difference suggests that, unlike oviparous and ovoviparous nematodes, whose embryonic development is completed based on the original provisioning of their eggs (lecithotrophy), embryos of *Tokorhabditis* n. gen. obtain some form of nutrition from their mother.

Although unusual among nematodes, obligate vivipary as described here has evolved convergently in the diplogastrid genus *Sudhausia*^17^. Intriguingly, *Tokorhabditis* n. gen. and *Sudhausia* are united by two similarities, despite vivipary being the result of two independent evolutionary events. First, both species have self-fertile hermaphrodites, and in *Sudhausia* this feature has gone to the extreme that the pre-adult stage is already sexually mature and can self-fertilize. Second, the ancestors of both genera were most likely associated with dung, i.e., of mammalian herbivores. Although *T.* *tufae* n. gen., n. sp. has thus far only been reported from Mono Lake, two other putative species of the new genus (represented by strains EJR13 and NKZ329) have been isolated from dung beetles, a finding that will be described elsewhere (Ragsdale and Kanzaki, in prep.). Although this similarity may be coincidence, it might also be speculated that dung somehow offers a habitat conducive to maternal investment through vivipary, a life-history strategy thought to offer protection from adverse environments^37^. For example, dung is known to host a diverse array of nematode (including predatory) species, some of which have clearly specialized for different growth curves during the cycle of dung decomposition^38^. It is thus possible that competition with, and threat of predation by, such cohabitants might have given such a pressure toward a viviparous lifestyle. The ability to empirically manipulate nematodes in the semi-natural setting of dung offers a route to testing this hypothesis^39^.

### Convergent reproductive morphologies correlating with obligate vivipary

Also concomitant with the convergent evolution of obligate vivipary in *Tokorhabditis* n. gen. and *Sudhausia* are striking similarities in vaginal morphology. Specifically, the female and hermaphrodite of *T.* *tufae* n. gen., n. sp. has a vagina comprising layers of stretched, elongated cells surrounding a Z-shaped vaginal duct. A similar morphology is found in *Sudhausia*, suggesting the morphology is functional in vivipary, for example to accommodate the passage of larger, developed juveniles while also being able to seal off the reproductive tract—that is, from environmental insults or natural pathogens—when not in use. Additionally, *Tokorhabditis* n. gen. shows another reproductive morphology—specifically, its vaginal fold—that is not found in either its sister group *Auanema* or in the distantly related, viviparous *Sudhausia*^10,17^.

### A comparative model for the evolution of trioecy

As previously described, *Auanema* comprises multiple species that have a trioecious mating system, which consists of males, females, and self-fertile hermaphrodites^10,30,40^. This mixed mating system has been considered very rare in animal systems^41^, and theory has predicted such a mating system to be transitional, i.e., between predominantly outcrossing and predominantly selfing modes of reproduction^42^. Here, we report a mating polymorphism similar to that of *Auanema* species, such that the eldest offspring of a sireless brood are males and females, followed by the emergence of self-fertile hermaphrodites. Because this form of trioecy is shared by both *Auanema* and *Tokorhabditis* n. gen., our report suggests that this mating system was ancestral to both genera, pushing back the presumptive evolutionary origin in rhabditid nematodes even further. Thus, we present a new point of comparison more precision in reconstructing the evolutionary correlates, for the evolution of this unusual mode of reproduction.

### A model for studying maternal care in nematodes

Maternal investment in nematodes, through mechanisms such as nutrient transport and yolk provisioning, can promote the survival of progeny in stressful environments^43^. Since *T.* *tufae* n. gen., n. sp. was isolated from the arsenic-rich environment of Mono Lake, we speculate that maternal investment in *Tokorhabditis* n. gen. was a preadaptation to the extreme stressors of that environment. Our description of this species offers an entry point to testing if, and to what degree, maternal care further facilitated an extremophilic lifestyle where the necessary physiological tolerance was already in place, since physiological tolerance of Mono Lake conditions are shared with *Auanema* species^8^. Our results suggest a trade-off between obligate vivipary and fecundity: selfing hermaphrodites of *T.* *tufae* n. gen., n. sp. produce an average of 115 progeny, which we found to be far less than the mean brood size of *C.* *elegans* as well as a representative of *Auanema* (*A.* *rhodensis*). The small brood size of *T.* *tufae* n. gen., n. sp. suggests a greater investment toward the development of individual progeny over the production of large broods in this species. However, we cannot rule out the possibility that the laboratory conditions used in this study were sub-optimal for *T.* *tufae* n. gen., n. sp. growth and thereby also reduced its brood size.

Clues to the mechanism of viviparity in *T.* *tufae* n. gen., n. sp. can be obtained from the mechanisms of maternal care in *C.* *elegans*. For instance, *C.* *elegans* mothers can secrete into their environment yolk milk that can be used to feed their young^44^. Milk production is controlled by insulin signaling and involves the breakdown of the gut to produce yolk, which contributes to senescence in the mother. This raises the question of whether nutrient transport in *T.* *tufae* n. gen., n. sp. viviparity involves similar signaling pathways and shortening of lifespan. Further studies of vivipary in *T.* *tufae* n. gen., n. sp. will be useful for comparing how nutrient transport and reproductive aging have diverged between this species and the well-characterized *C.* *elegans*.

## Supplementary Information

Supplementary Video 1.

Supplementary Video 2.

Supplementary Legends.

Supplementary Table S1.
