# Supplementary material for: Tokorhabditis n. gen. (Rhabditida, Rhabditidae), a comparative nematode model for extremophilic living
Source: Sci Rep. 2021 Aug 13;11:16470. doi: 10.1038/s41598-021-95863-1 (PMC8363662; doi:10.1038/s41598-021-95863-1)
Supplement: Supplementary file 3 — Supplementary Legends. [file 41598_2021_95863_MOESM3_ESM.docx]

Legends for supplemental Data

**Supplementary Data 1.** Giving birth of *Tokorhabditis tufae* n. gen., n. sp. hermaphrodite. A juvenile actively moving inside the uterus finds uterus-vagina junction, and quickly goes out from the mother nematode through vagina.

**Supplementary Data 2.** Movement of dauer juveniles of *Tokorhabditis tufae* n. gen., n. sp. The dauer juveniles of the species actively crawl around the substrate (NGM agar in this case) without showing tube-waving behavior on the substrate surface.
