# Supplementary material for: Tokorhabditis n. gen. (Rhabditida, Rhabditidae), a comparative nematode model for extremophilic living
Source: Sci Rep. 2021 Aug 13;11:16470. doi: 10.1038/s41598-021-95863-1 (PMC8363662; doi:10.1038/s41598-021-95863-1)
Supplement: Supplementary file 4 — Supplementary Table S1. [file 41598_2021_95863_MOESM4_ESM.docx]

**Supplementary Table S1.** Accession numbers for sequences in the phylogenetic analysis.

| Species | Accession number |
| --- | --- |
| *Tokorhabditis tufae* n. gen., n. sp. | LC639824 |
| *Tokorhabditis* sp. NKZ329 | LC639823 |
| *Tokorhabditis* sp. EJR13 | LC639822 |
| *Auanema freiburgensis* | KY680647 |
| *Auanema rhodensis* | EU196004 |
| *Cephaloboides* sp. | AF083027 |
| *Haematozoon subulatum* | AF083017 |
| *Heterorhabditis bacteriophora* | FJ040428 |
| *Litoditis marina* | AF083021 |
| *Litoditis mediterranea* | AF083020 |
| *Mesorhabditis rainai* | AF083008 |
| *Oscheius carolinensis* | FJ547240 |
| *Oscheius dolichura* | EU196010 |
| *Oscheius dolichuroides* | AF082998 |
| *Oscheius guentheri* | EU196022 |
| *Oscheius insectivora* | AF083019 |
| *Oscheius myriophilus* | KP756941 |
| *Oscheius tipulae* | KP756939 |
| *Oscheius* sp. BW282 | AF082994 |
| *Phasmarhabditis* sp. EM434 | EU196008 |
| *Rhabditella axei* | AY284654 |
| *Rhabditella* sp. DF5044 | AF083000 |
| *Rhabditis blumi* | U13935 |
| *Rhabditis brassicae* | EU196006 |
| *Rhabditis* cf. *terricola* | AY284653 |
| *Rhabditis typica* | U13933 |
| *Rhabditis* sp. DF5059 | EU196007 |
| *Rhabditis* sp. RA5 | HQ130504 |
| *Rhabditoides inermis* | AF082996 |
| *Rhomborhabditis regina* | AF082997 |

Sequences were selected for analysis as informed by a previous study ^10^.
